# A Phase-Shifting Method for Improving the Heating Uniformity of Microwave Processing Materials

**DOI:** 10.3390/ma9050309

**Published:** 2016-04-25

**Authors:** Yinhong Liao, Junqing Lan, Chun Zhang, Tao Hong, Yang Yang, Kama Huang, Huacheng Zhu

**Affiliations:** 1 College of Electronic and Information Engineering, Sichuan University, Chengdu 610065, China; liaoyinhong2006@126.com (Y.L.); chun-199012@163.com (C.Z.); scu_mandela@163.com (T.H.); yyang@scu.edu.cn (Y.Y.); kmhuang@scu.edu.cn (K.H.); 2 College of Electronic Engineering, Chengdu University of Information Technology, Chengdu 610065, China; jqlan@cuit.edu.cn

**Keywords:** materials processing, microwave heating, sliding short, transformation optics

## Abstract

Microwave processing of materials has been found to deliver enormous advantages over conventional processing methods in terms of mechanical and physical properties of the materials. However, the non-uniform temperature distribution is the key problem of microwave processing, which is related to the structure of the cavity, and the placement and physical parameters of the material. In this paper, a new microwave cavity structure with a sliding short based on phase-shifting heating is creatively proposed to improve the temperature uniformity. An electronic mathematical model based on the Finite Element Method (FEM) is built to predict the temperature distribution. Meanwhile, a new computational approach based on the theory of transformation optics is first provided to solve the problem of the moving boundary in the model simulation. At first, the experiment is carried out to validate the model, and heating results from the experiment show good agreement with the model’s prediction. Based on the verified model, materials selected among a wide range of dielectric constants are treated by stationary heating and phase-shifting heating. The coefficient of variation (COV) of the temperature and temperature difference has been compared in detail between stationary heating and phase-shifting heating. A significant improvement in heating uniformity can be seen from the temperature distribution for most of the materials. Furthermore, three other materials are also treated at high temperature and the heating uniformity is also improved. Briefly, the strategy of phase-shifting heating plays a significant role in solve the problem of non-uniform heating in microwave-based material processing. A 25%–58% increase in uniformity from adapting the phase-shifting method can be observed for the microwave-processed materials.

## 1. Introduction

Microwave energy has become a newly-developing energy source in recent years. Since its first application reported by Roy *et al.* in 1990, microwave heating technology and related equipment have been widely applied in both industrial and small-scale applications, such as the microwave sintering of metal and alloy, and the processing of food, ceramics, composites, biomaterials, chemical and agricultural products [1,2,3,4,5,6,7,8]. Many potential advantages have been recognized in the area of materials processing. Microwaves heat the material from the inside and can lead to saving enormous amounts of energy since it is not necessary to heat the container or the air between the heat source and the load material. Microwave processing can facilitate the densification rate and particle size uniformity and can also promote improvement in microstructural properties of materials which cannot be observed in conventional processes.

Currently, the microwave processing of materials is at the take-off stage and many successful uses have been achieved in the production of materials. For example, Roy *et al.* demonstrated that the modulus of Fe–Ni rupture is 60% higher than that of conventional samples after 10–30 min of microwave processing [5]. Janney *et al.* confirmed that the activation energy in microwave sintering is lower compared with that in conventional sintering [6]. Saha *et al.* successfully utilized microwaves in agglomerating dental ceramics [7]. P. Nagaraja Upadhya applied microwave irradiation to the melting of calcium fluoroaluminosilicate glasses and compared the structure of the glasses processed by the conventional melt-quench route. The results show that microwave-assisted melting promoted more uniform degradation of the glass, resulting in cement forming cations at a faster rate. Meanwhile, microwaves had not altered the composition of the glass and microwave melt-processed glasses and conventional melted cements did not reveal significant variation in their properties [8].

Unfortunately, some limitations restrict the up-scaling of laboratory-based experiments to industrial application. Microwave heating is liable to cause non-uniform temperature distribution. The hot spots (huge temperature gradient at a given location) and thermal runaway (the uncontrollable temperature rise due to strong dielectric loss and temperature-positive feedback of the material) caused by microwave non-uniform heating may occur when a high-power microwave is applied on the processed materials. In some instances, it even leads to the damage of material or even an explosion. Obviously, the problem of non-uniform heating has become a bottleneck for the widespread exploitation of this technology.

In previous work, there have been large numbers of studies about improving the uniformity of microwave heating. The research carried out by Funawatashi and Suzuki came to the conclusion that non-uniform heating was due to the standing wave and the rapid decay of the microwave. Based on the analysis, they proposed that metallic stirrers and turning tables employed in microwave ovens can reduce the degree of non-uniform microwave heating [9]. Another research article by Pedreño-Molina *et al.* focused on the optimal strategy of movement of the sample placed in the microwave applicator to ensure better uniform heating [10,11]. Additionally, a mode stirrer was implied to change the electric field distributions and the mode of the electromagnetic field in multi-mode cavities, achieving more uniform electric field distribution [12,13,14]. Another study by Bows proposed phase shifting as a novel method of microwave heating, and indicated that uniform heating can be promoted when samples are heated in various phase differences by changing the position of samples inside particular microwave applicators [15].

In order to solve the non-uniform heating problem, a novel, efficient and simplified structure is first proposed. This structure is based on the theory of phase shifting but is totally different from other cavity structures. The cavity structure is efficient, relatively inexpensive and user-friendly. In this article, the metal boundary of the waveguide cavity (called the sliding short) was moved at a certain velocity, and thus the distribution of the electromagnetic field is dynamically changed, leading to a more uniform distribution in the material and the cavity during the microwave processing. To validate the effect of the structure on the improvement of uniform heating, at first the COMSOL software based on the Finite Element Method (FEM) was employed to build the electronic mathematical model for the multi-physics simulation of microwave heating. Meanwhile, the theory of transformation optics was first introduced to overcome the difficulty of multi-physics calculations for a moving boundary. Thereafter, an elaborate experiment was designed to measure some point temperature histories and spatial temperature distribution, which are compared with model predictions at the same time. The experimental results obtained from the infrared camera and optical thermometer verified that the model and computational results are reliable. In order to demonstrate that the phase-shifting method can promote the apparent improvement of temperature uniformity for processing most types of materials, two other materials (NaCl, methanol) with a broad range of permittivity were included in the simulation. The experimental results have shown that phase shifting can be used to improve the heating uniformity for most types of materials. Finally, the application of phase shifting for material processing was also discussed at a high temperature. Graphite, ZnO and SiC were processed at a high temperature level (about 500 °C). The experimental results also showed that phase shifting can improve the heating uniformity of materials at high temperature.

## 2. Methodology

### 2.1. Model Description

**Figure 1:**
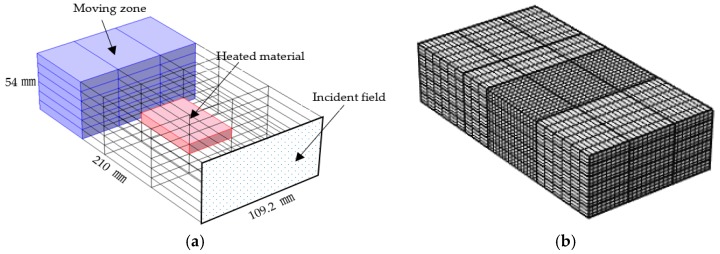
Geometry (**a**) and finite element mesh (**b**) of the microwave cavity in this study.

The microwave oven model used in the article consists of a simple waveguide cavity containing a block of material, as shown in Figure 1a. One side of the cavity is set as a scattering boundary and connected to a microwave source. The field in the cavity is excited by a transverse electric (TE) wave, which is a wave that has no electric field component in the direction of propagation. Except the face that is set as a scattering boundary condition, others are assumed as perfect electric conductors. When the model is divided into several rectangle domains, three-dimensional (3D) swept meshes (sweeping the mesh from the source face along the domain to the opposite face) can be employed in a mesh building, as shown in Figure 1b.

Table 1 lists the input parameters of the model. Potato is chosen as the material sample for microwave heating. Considering the narrow temperature range, the thermal property and dielectric constant are treated as constant in this experiment.

### 2.2. Multi-Physics Calculation

Microwave-assisted processing of materials involves multiple physics, electromagnetic in the cavity and heated samples, as well as mass and heat transport.

#### 2.2.1. Governing Equation

For the multi-physics calculation of microwave processing of materials, the electromagnetics and fluid heat transfer are coupled. The basic equations describing the electromagnetic field distribution inside the microwave cavity are Maxwell equations:

(1) $$\begin{array}{l} \nabla \times \vec{H}=\frac{\partial \vec{D}}{\partial t}+\vec{J}\\ \nabla \times \vec{E}=-\frac{\partial \vec{B}}{\partial t}\\ \nabla \cdot \vec{D}=\rho \\ \nabla \cdot \vec{B}=0 \end{array}$$

where $\vec{E}$ and $\vec{H}$ are the electric field and magnetic field vector, $\vec{D}$ is the electric displacement vector, $\vec{B}$ is the magnetic flux density vector and $\vec{J}$ is the conductive current density vector. The distribution of the electric field can be solved by the above equations, and the power dissipation of per unit volume can be calculated by:

(2) $$P_{d}(\vec{r},t)=\frac{1}{2}(\vec{E}\cdot \frac{\partial \vec{D}}{\partial t}-\vec{D}\cdot \frac{\partial \vec{E}}{\partial t})+\vec{J}\cdot \vec{E}$$

The temperature rise in the reaction system is given by Fourier’s Law:

(3) $$\rho_{m}C_{p}\frac{\partial T(\vec{r},t)}{\partial t}=K_{t}\nabla^{2}T(\vec{r},t)+P_{d}(\vec{r},t)$$

where $\rho_{m}$, $C_{p}$ and $K_{t}$ are, respectively, the equivalent medium density, specific heat capacity and thermal conductivity of the medium.

#### 2.2.2. Boundary Condition

- Electromagnetic boundary condition

The tangential components of the electric field and magnetic field must be continuous, especially across the boundary. For microwave oven, the metallic cavity is regarded as a perfect electrical conductor, the fields inside the conductor vanish and the tangential components of the electric field are zero on the boundary. Mathematically, we write:

(4) $$\hat{n}\times \vec{E}=0$$

(5) $$\hat{n}\cdot \vec{B}=0$$

where $\hat{n}$ is the unit vector perpendicular to the interface.

- Heat transfer boundary condition

The boundary condition of heat transfer describes the thermal convection of the temperature field at the interface. Usually, the interfacial heat loss due to air flow is modeled by specifying the convective heat transfer coefficient and temperature of a random point M on interface S:

(6) $${k_{n}(\frac{\partial T}{\partial n})|}_{MeS}+h(T_{S_{3}}-T_{a})=0$$

where $k_{n}$ is the thermal conductivity perpendicular to the interface, $\frac{\partial T}{\partial n}$ is the gradient of the temperature perpendicular to the temperature field interface, *h* is the convection heat transfer coefficient over a surface, $T_{a}$ is the temperature of the air inside the oven and $T_{S}$ is the temperature of S on the interface.

### 2.3. The Transformation Optics Algorithms for Sliding Short

In the detailed structure design of the microwave cavity and in the optimization of the sample movement strategy, a large number of design variables must be calculated, but the moving boundary of this system makes the calculation process become complex. In order to simplify the computing, the theory of transformation optics is applied to the coordinate transformation. It can be used to reduce the calculation size of the embedded source. The coordinate transformation is defined as:

(7) $$\vec{r}\to {\vec{r}}^{\prime }=T\left(\vec{r}\right)$$

where $\vec{r}$ and ${\vec{r}}^{\prime }$ are the vectors of the positions in the original and present coordinate system, respectively. Especially the form of the Maxwell equations remains the same, even in different coordinate system. So the permittivity and permeability can be written as [19]

(8) $$\bar{\bar{\varepsilon }}=\varepsilon \bar{\bar{\Lambda }}$$

(9) $$\bar{\bar{\mu }}=\mu \bar{\bar{\Lambda }}$$

(10) $$\bar{\bar{\Lambda }}=(\det\bar{\bar{J}})\cdot {\left({\bar{\bar{J}}}^{T}\cdot \bar{\bar{J}}\right)}^{-1}$$

where ε and μ are the permittivity and permeability in the original coordinate system; $\bar{\bar{J}}$ is Jacobi’s tensor, which is defined as:

(11) $$\bar{\bar{J}}=\left[\begin{array}{ccc} \partial x^{\prime }/\partial x & \partial x^{\prime }/\partial y & \partial x^{\prime }/\partial z\\ \partial y^{\prime }/\partial x & \partial y^{\prime }/\partial y & \partial y^{\prime }/\partial z\\ \partial z^{\prime }/\partial x & \partial z^{\prime }/\partial y & \partial z^{\prime }/\partial z \end{array}\right]$$

The electric and magnetic field in two coordinate systems can be expressed by Jacobi’s tensor [20].

(12) $$\vec{E}\left(\vec{r}\right)={\left({\bar{\bar{J}}}^{T}\right)}^{-1}\cdot \tilde{\vec{E}}\left({\vec{r}}^{\prime }\right)$$

(13) $$\vec{H}\left(\vec{r}\right)={\left({\bar{\bar{J}}}^{T}\right)}^{-1}\cdot \tilde{\vec{H}}\left({\vec{r}}^{\prime }\right)$$

**Figure 2:**
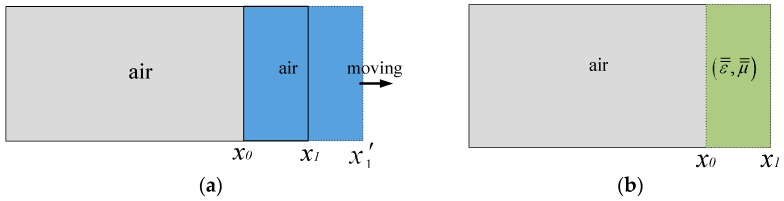
Schematic diagram of transformation optics: (**a**) Description of moving waveguide wall; (**b**) Description equivalent result of coordinate transformation.

According to coordinate transformation, the change in position of the sliding short from *x*_1_ to *x*_1_’ can be equally expressed by substituting $\bar{\bar{\varepsilon }}$ and $\bar{\bar{\mu }}$ for ε and μ, as shown in Figure 2. So the movement of the sliding short is equivalent to altering the related physical parameters of the moving zone in Figure 1a. In this way, we can solve the problem of simulating the moving boundary with the help of multi-physics software COMSOL Multiphysics (COMSOL Inc., Stockholm, Sweden).

### 2.4. Phase-Shifting Method and Experiment Setup

#### 2.4.1. Phase-Shifting Method

**Figure 3:**
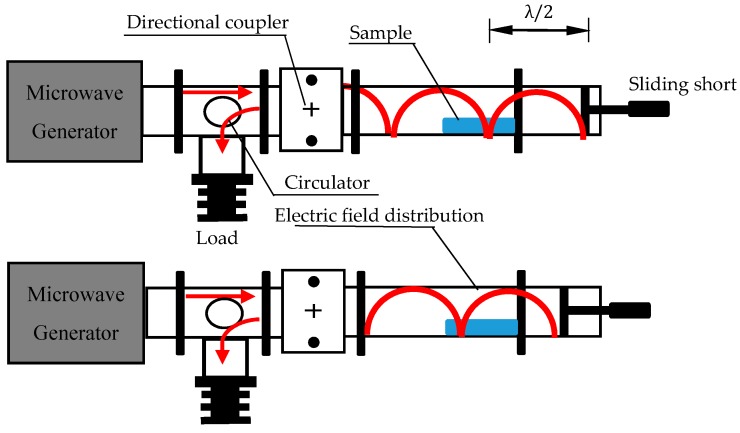
A schematic diagram of the effect of moving the sliding short on the electric distribution.

The electrical field pattern located in the waveguide (called mode) usually varies for different structures of the cavity and the characters of processing materials. Figure 3 demonstrates how the sliding short (connected to the end of the waveguide) is exploited to change the spatial distribution of the microwave field in the BJ-22 waveguide system. As the terminal of the cavity is shorted, only standing waves exist in the waveguide. The position of the sliding short is an electric field node, λ/4 away from an antinode. The node and antinode reappear every other λ/2. Therefore, the distribution of the electric field in the cavity and the amplitude of the electric field applied to the sample can be changed by moving the position of the sliding short. In this way, it is then possible to generate different intensities of electric field in the position of the sample, avoiding hot or cold spots resulting from non-uniform static electric field distribution.

#### 2.4.2. Experiment Setup

**Figure 4:**
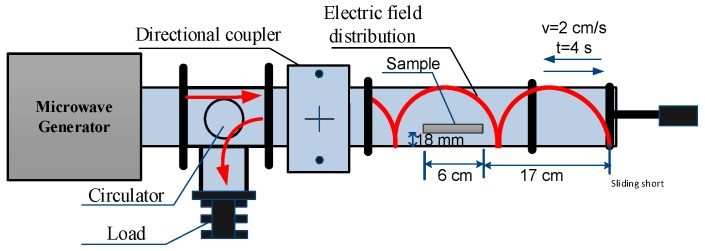
Experiment setup of microwave processing of material sample.

Figure 4 demonstrates the experiment setup. The system consists of a microwave generator, circulator, waveguide and sliding short. Microwave energy travels from the generator to the circulator, the coupler and then radiates into the cavity, and the reflection of the energy can be absorbed by the microwave load via the circulator, avoiding damaging the generator. In the study on the effect of the sliding short on the microwave heating uniformity, the sliding short moved backwards and forwards in the horizontal direction at the speed of 2 cm/s with the microwave power on. The period of the sliding short moving from side to side is 4 s. The BJ-22 waveguide system operating at 2450 MHz and the TE10 mode is excited in the waveguide. The sample is exposed to a microwave with an output power of 700 W. The output power is determined by using water as the load of the cavity and measuring the microwave heating time for a constant rise in the temperature of water [21]. The point temperature on the center line of the upper surface was measured during the heating process by a fiber optic system (Model: FISO Technology Inc. 500 UMI-8, Bremen, Germany). A thermal camera (Type: Infertech VarioCam HiRes, JENOPTIK, Jena, Germany) was applied to get the surface temperature distribution of the heated sample as soon as it came out from the microwave cavity.

**Figure 5:**
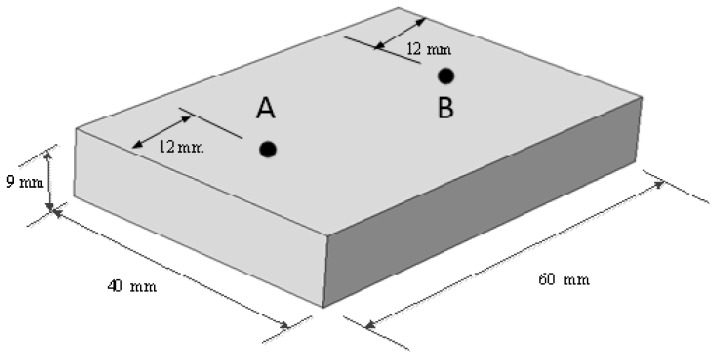
The aperture size of heated sample.

Owing to electric field distribution repeating regularly every other λ/2 (6.13 cm), to ensure the sample is heated during the whole process of phase shifting, the movement distance of the sliding short going backwards and forwards is set as 8 cm (2 cm/s × 4 s). The material sample, potato (approximate volume: 40 mm × 60 mm × 9 mm), as shown in Figure 5, is placed 23 cm away from the right end of the waveguide, 18 mm from the bottom.

## 3. Results and Discussion

### 3.1. Experimental Validation

In order to validate the computational model, at first the experimental measured temperature histories of the sample at different locations of the upper surface are compared to the calculated results obtained from the model. Meanwhile, temperature distributions calculated by two methods are compared with experimental measured results.

**Figure 6:**
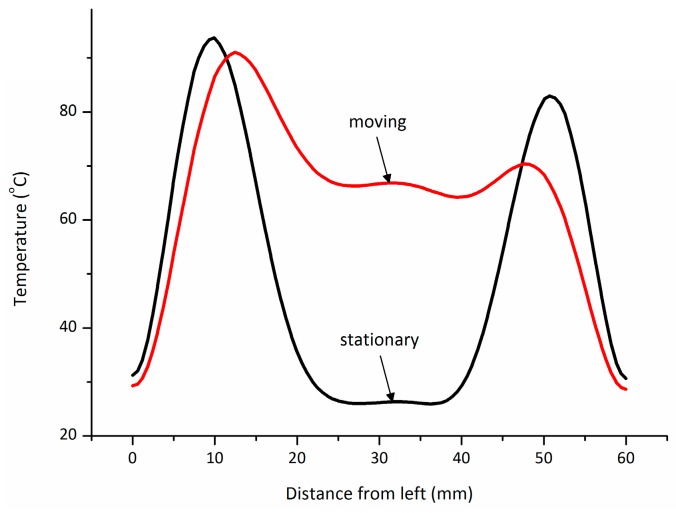
Comparison of temperature on the center of the surface between two heating treatments.

**Figure 7:**
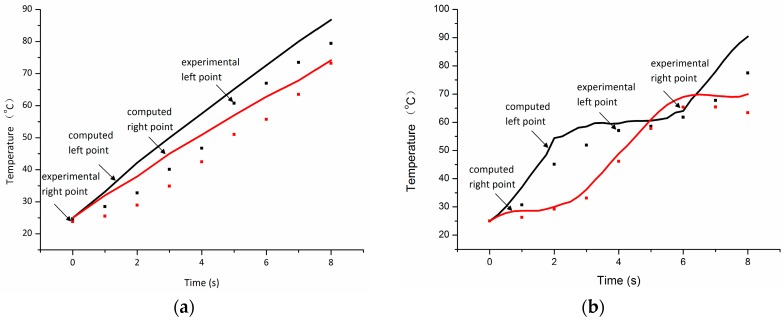
Computed and experimental temperature histories at point A and B (in Figure 5) without (**a**) and with (**b**) moving of sliding short.

Figure 6 and Figure 7 present the measured and calculated temperature histories on the center of surface taken by an optical fiber thermometer. Because of the quick heat dissipation in air, the experimental temperature of the sample is about 15 °C lower than the simulation result. Comparing Figure 7a with Figure 7b, the distributions obtained from model and experiment do not show a significant difference for different methods. Hence, the accuracy of the model can be ascertained. Furthermore, it is obvious that the sliding short has great influence on the uniform distribution of temperature.

**Figure 8:**
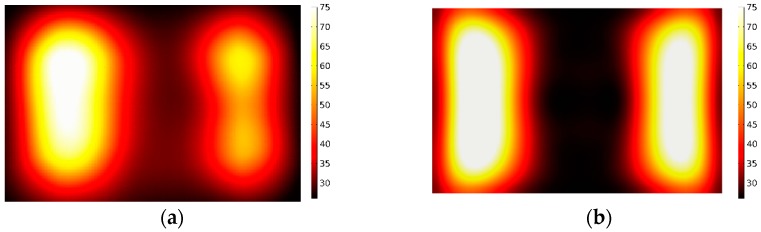
Comparison of the calculated and measured temperature distribution in stationary heating: (**a**) Measurement; (**b**) Simulation.

**Figure 9:**
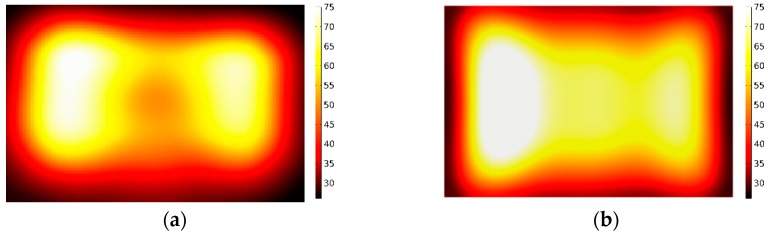
Comparison of the calculated and measured temperature distribution in moving heating: (**a**) Measurement; (**b**) Simulation.

Two kinds of different microwave treatments applied to the sample are carried out separately by fixing and moving the sliding short during the entire process of microwave heating. For the former treatment, the sliding short is fixed to stay 15 cm away from the left edge of the sample. For the latter one, the sliding short is swung within the distance of 8 cm at a moving velocity of 2 cm/s. In thermal imaging, the dark and bright colors represent, respectively, low and high temperature. The measured and simulated temperature distribution of the surface, obtained from the infrared thermal camera using two methods, are shown in Figure 8 and Figure 9. The experimental results are found to match with the calculated values.

### 3.2. Results Discussion

- Different kinds of materials

**Figure 10:**
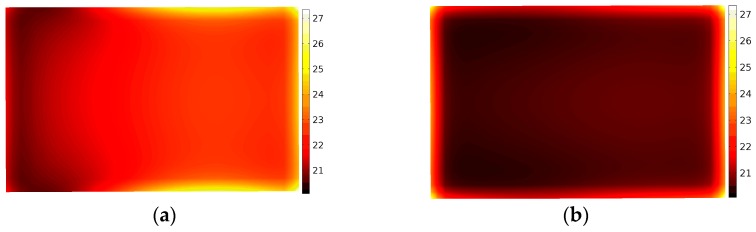
Comparison of temperature distribution of NaCl without (**a**) and with (**b**) moving of the sliding short.

**Figure 11:**
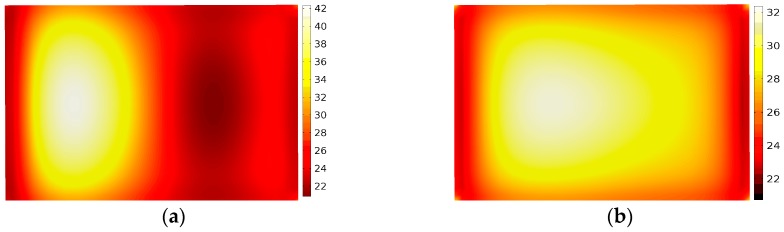
Comparison of temperature distribution of methanol without (**a**) and with (**b**) moving of the sliding short.

In order to ensure that the method of phase shifting is useful in uniform heating for the majority of materials, we substitute two other kinds of material (NaCl, methanol) for the potato in the further study. As the model has been validated, we can employ this model speculation to estimate the influence of phase shifting on the improvement of uniform heating for other materials. Figure 10 and Figure 11 demonstrate the surface temperature distribution of the samples at the end time of being treated by static heating and the phase-shifting method. More uniform distribution can be seen in Figure 10b and Figure 11b.

**Figure 12:**
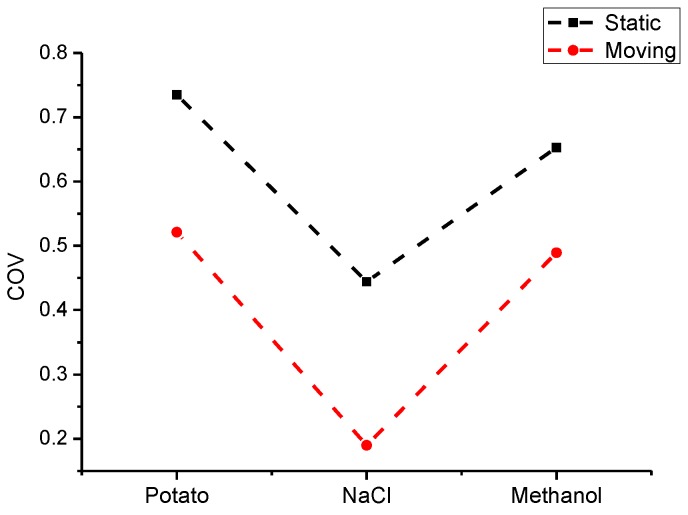
Comparison of COV for different materials in static and moving heating.

**Figure 13:**
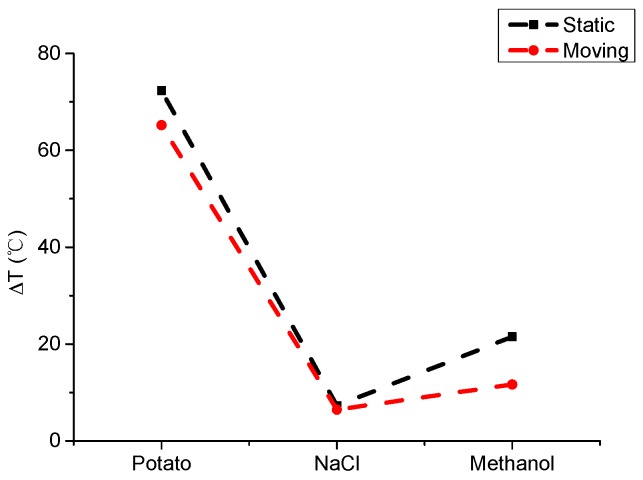
Comparison of temperature difference for different materials in static and moving heating.

To accurately express the degree of improvement on the uniformity of temperature, the coefficients of variation (COV) of the temperature and temperature difference for different materials are used as the identifiers for the temperature uniformity, as shown in Figure 12 and Figure 13.

The COV is the ratio of the standard deviation to the mean. The COV of the potato in stationary heating is 0.735 and that of moving heating is 0.521. This can be considered as a (0.735–0.521)/0.735 or 29% increase in temperature uniformity. For NaCl and methanol, there will be 57% and 25% increases. On the other hand, the temperature difference can directly reflect the range of temperatures. From Figure 12 and Figure 13, a significant improvement of uniformity can be seen for three samples, especially for NaCl with a low permittivity. For the methanol and potato, the effect of improvement is not evident but it is still useful to avoid a hot spot of the temperature.

Considering the large-scale range of the dielectric constant of three kinds of materials (from 5.2 to 70), to a certain extent they can represent the majority of materials. Hence, the phase-shifting method can ensure better temperature uniformity for the overwhelming majority of materials (especially for those with low permittivity).

- The application of phase shifting for material processing

**Figure 14:**
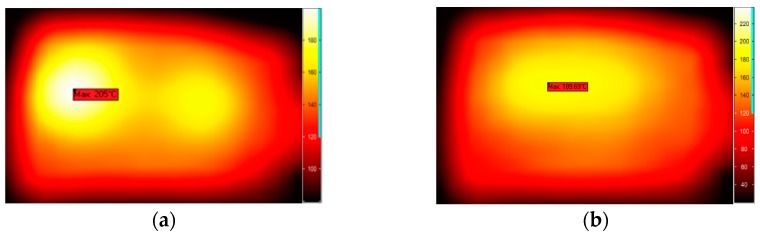
The temperature distribution of graphite in stationary heating (**a**) and moving heating (**b**).

**Figure 15:**
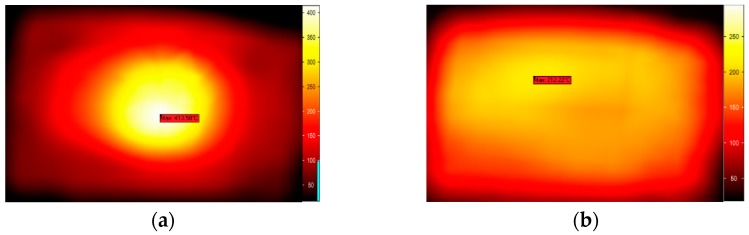
The temperature distribution of ZnO in stationary heating (**a**) and moving heating (**b**).

**Figure 16:**
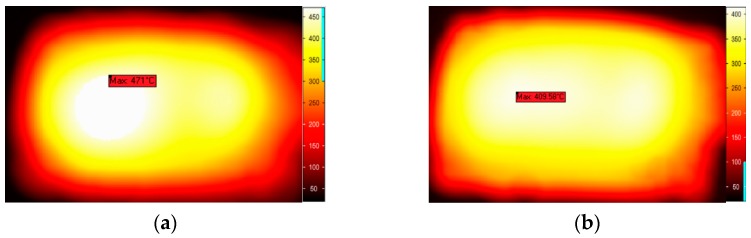
The temperature distribution of SiC in stationary heating (**a**) and moving heating (**b**).

In a previous study, potato, NaCl and methanol were applied to an experimental study at a low temperature (below 100 °C), and theoretically, the method has general applicability in material processing. In this section, the application of phase shifting for material processing is discussed at a high temperature. Graphite, ZnO and SiC were processed by microwave (with the output power of 700 W, processing time of 12 min), respectively. After two different treatments, the temperature distributions were compared in Figure 14, Figure 15 and Figure 16.

These figures give a very good qualitative description of the effect of the sliding short on the microwave processing of material temperature uniformity. In the case of moving heating, the temperature contours show a more uniform pattern on the surface whereas the stationary heating samples have a region of concentrated high temperature. The phase-shifting method can effectively avoid the hot spot induced in the traditional method. So the same conclusion can be drawn that the phase-shifting method can ensure a better uniform temperature distribution in the microwave processing of materials, even with a heating temperature up to 500 °C.

## 4. Conclusions

In this paper, we have proposed a new kind of method to solve the non-uniform heating problem of the microwave processing of materials, and we apply the theory of transformation optics to the multi-physics calculation for the moving boundary. The computed results were compared with the measured ones, which indicate that the method of phase shifting can make a great contribution in the improvement of uniform temperature distribution.

## Figures and Tables

**Table 1:** Input parameters used in the simulation.

Parameter	Value	Source
Cavity interior dimensions (mm)	109.2 × 210 × 54	This study
Sample dimension (mm)	40 × 60 × 9	This study
Placement of material position (mm) (center)	54.6 (*x*) 90 (*y*) 22.5 (*z*)	This study
Microwave frequency (MHz)	2450	This study
Heating time (s)	8	This study
Dielectric constant and loss of potato	70-j18 (15 °C)	Gulati, T. [16]
Dielectric constant and loss of NaCl	5.20-j10.61 (15 °C)	Chen, Q [17]
Dielectric constant and loss of methanol	25.910-j14.523 (15 °C)	Chen, Q [18]
Microwave frequency (GHz)	2.45	This study
Microwave power (W)	700	This study
